# Supplementary material for: Pharmacophore Mapping Combined with dbCICA Reveal New Structural Features for the Development of Novel Ligands Targeting α4β2 and α7 Nicotinic Acetylcholine Receptors
Source: Molecules. 2022 Nov 25;27(23):8236. doi: 10.3390/molecules27238236 (PMC9735964; doi:10.3390/molecules27238236)

SUPPLEMENTARY MATERIAL

Pharmacophore Mapping Combined with dbCICA Reveal New Structural Features for the Development of Novel Ligands Targeting α4β2 and α7 Nicotinic Acetylcholine Receptors

Summary

Mathematical Formula S1 - Mathematical formula to calculate Euclidean distance between the studied ligands. 2

Supplementary Figure S1 – Ramachandran plot of $(\alpha4)_2(\beta2)_3$ (PDB 5KXI) (a) and α7 nAChRs (PDB 5afh) (b) structures. 3

Supplementary Figure S2 – Chemical structures of the 98 ligands used to construct the dbCICA models for the $(\alpha4)_2(\beta2)_3$ subtype nAChRs. 4

Supplementary Figure S3 - Chemical structures of the 93 ligands used to construct the dbCICA models for the α7 subtype nAChRs. 7

Supplementary FIGURE S4 – ROC curves of $(\alpha4)_2(\beta2)_3$ (a) and α7 (b) nAChRs pharmacophore maps validation. 11

SUPPLEMENTARY MATERIAL

Euclidian distance is based on a calculus involving the number of hydrogen acceptor and donor atoms and heavy atoms of each molecule, the resulting value of each molecule is compared to that of all the others.

Mathematical formula S1. - Mathematical formula to calculate Euclidean distance between the studied ligands.

$$D(i,j) = \sqrt{(HBDi - HBDj)^2 + (HBAi - HBDj)^2 + (NPi - NPj)^2}$$

HBD = Hydrogen bond donor atoms

HBA = Hydrogen bond acceptor atoms

NP = Non-polar heavy atoms

According to Ramachandran plot both nAChRs structures (α4β2 - PDB 5KXI and α7 - PDB 5AFH) have at least 89% of their residues in favored regions and 10% in allowed regions. None of the amino acid residues used in the study are in generously allowed or disallowed regions.

Figure S1. – Ramachandran plot of $(\alpha4)_2(\beta2)_3$ (PDB 5KXI) (a) and α7 nAChRs (PDB 5AFH) (b) structures.

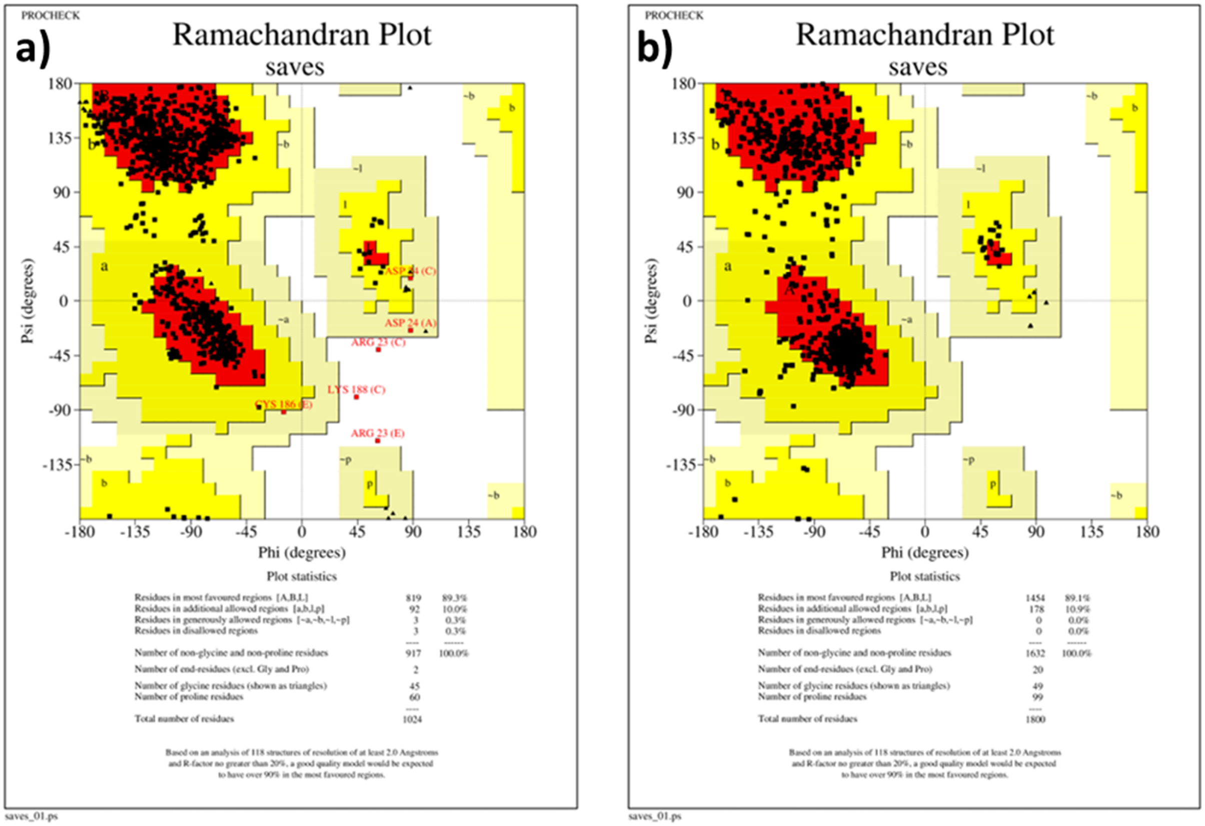

All 98 ligands structures and their K_i used for dbCICA analysis of $(\alpha4)_2(\beta2)_3$ are shown in Supplementary figure 2.

Figure S2. – Chemical structures of the 98 ligands used to construct the dbCICA models for the $(\alpha4)_2(\beta2)_3$ subtype nAChRs.

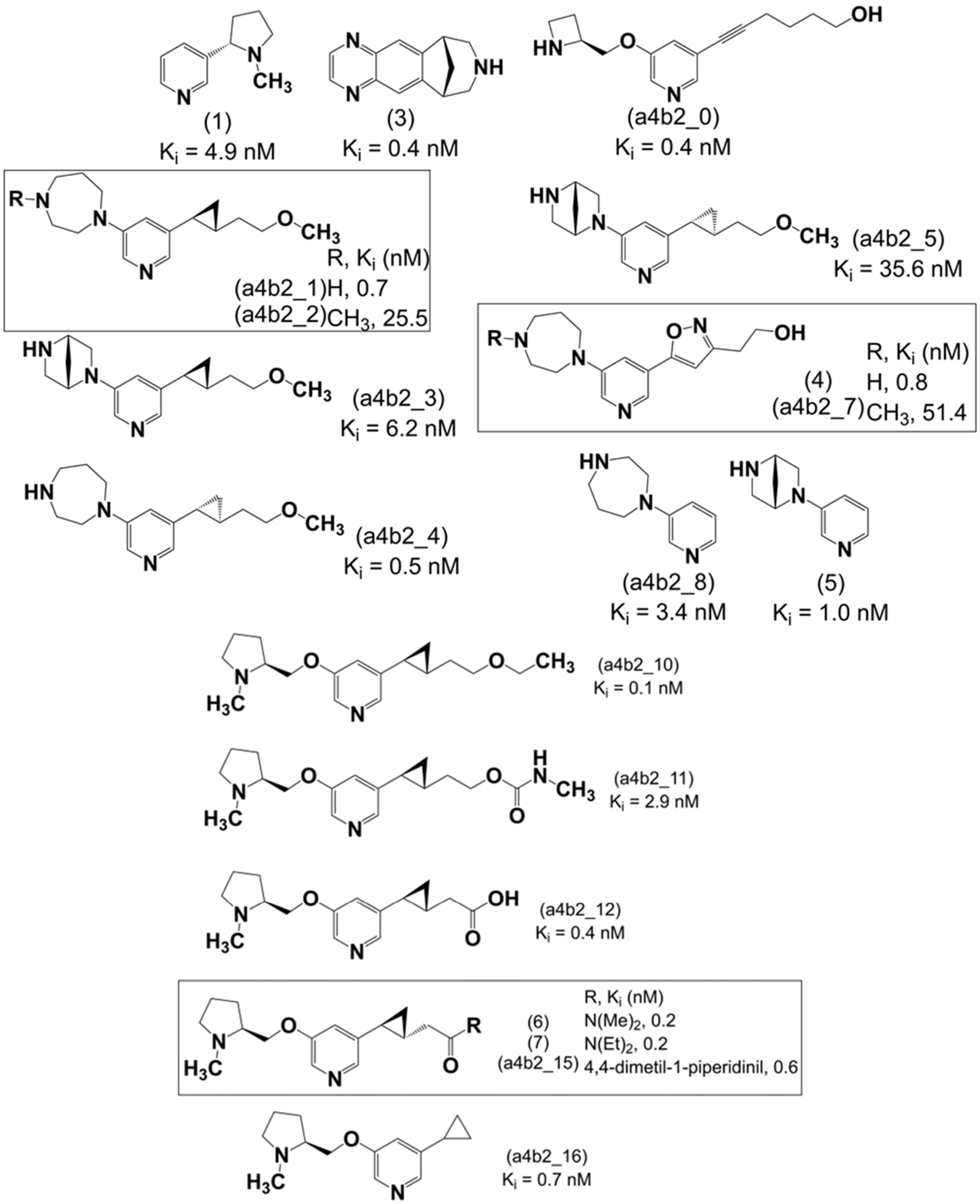

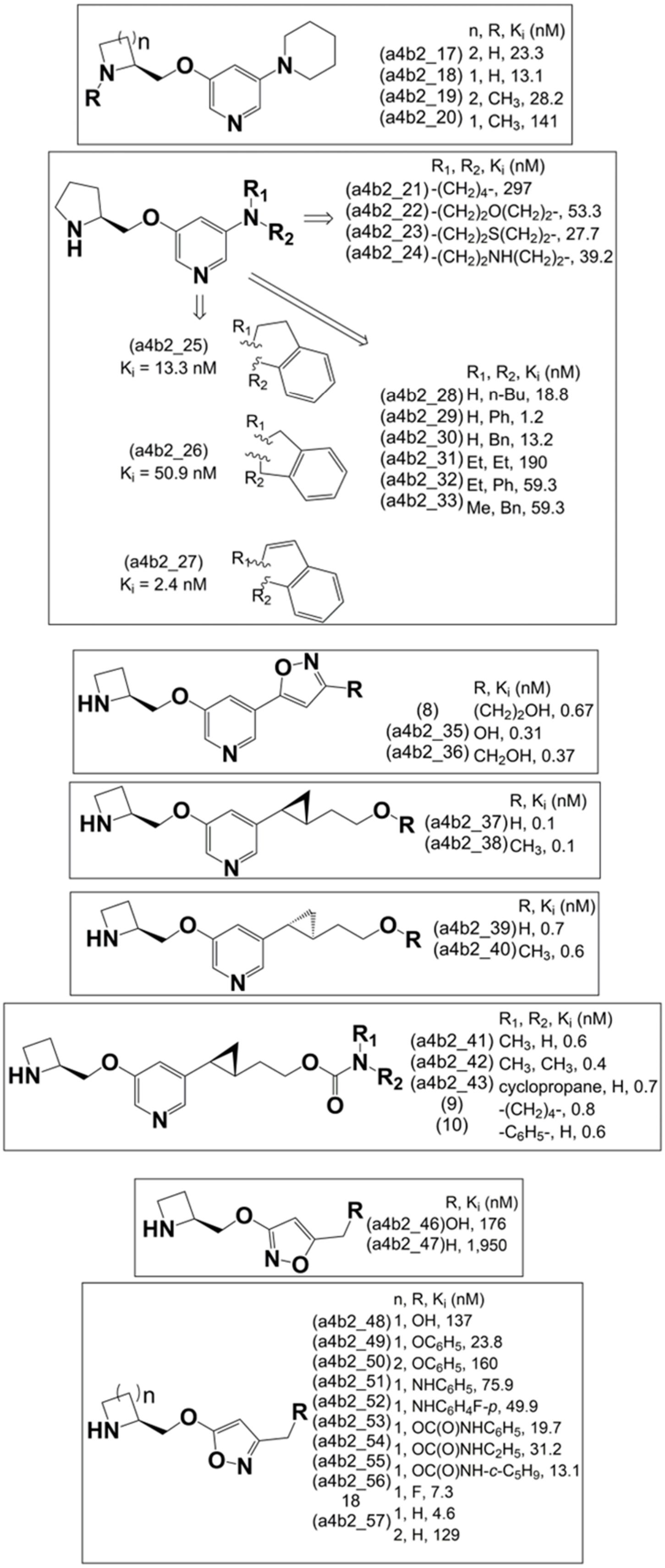
n
N
R
O
N
N
n, R, K_i (nM)
(a4b2_17) 2, H, 23.3
(a4b2_18) 1, H, 13.1
(a4b2_19) 2, CH_3, 28.2
(a4b2_20) 1, CH_3, 141
N
H
O
N
R_1
N
R_2
R_1, R_2, K_i (nM)
(a4b2_21) -$(CH_2)_4$-, 297
(a4b2_22) -$(CH_2)_2O(CH_2)_2$-, 53.3
(a4b2_23) -$(CH_2)_2S(CH_2)_2$-, 27.7
(a4b2_24) -$(CH_2)_2NH(CH_2)_2$-, 39.2
(a4b2_25)
K_i = 13.3 nM
R_1
R_2
(a4b2_26)
K_i = 50.9 nM
R_1
R_2
(a4b2_27)
K_i = 2.4 nM
R_1
R_2
R_1, R_2, K_i (nM)
(a4b2_28) H, n-Bu, 18.8
(a4b2_29) H, Ph, 1.2
(a4b2_30) H, Bn, 13.2
(a4b2_31) Et, Et, 190
(a4b2_32) Et, Ph, 59.3
(a4b2_33) Me, Bn, 59.3
HN
O
N
O-N
R
R, K_i (nM)
(8) $(CH_2)_2OH$, 0.67
(a4b2_35) OH, 0.31
(a4b2_36) CH_2OH, 0.37
HN
O
N
O-R
R, K_i (nM)
(a4b2_37) H, 0.1
(a4b2_38) CH_3, 0.1
HN
O
N
O-R
R, K_i (nM)
(a4b2_39) H, 0.7
(a4b2_40) CH_3, 0.6
HN
O
N
O
O
R_1
N
R_2
R_1, R_2, K_i (nM)
(a4b2_41) CH_3, H, 0.6
(a4b2_42) CH_3, CH_3, 0.4
(a4b2_43) cyclopropane, H, 0.7
(9) -$(CH_2)_4$-, 0.8
(10) -C_6H_5-, H, 0.6
HN
O
N-O
R
R, K_i (nM)
(a4b2_46) OH, 176
(a4b2_47) H, 1,950
n
HN
O
O-N
R
n, R, K_i (nM)
(a4b2_48) 1, OH, 137
(a4b2_49) 1, OC_6H_5, 23.8
(a4b2_50) 2, OC_6H_5, 160
(a4b2_51) 1, NHC_6H_5, 75.9
(a4b2_52) 1, NHC_6H_4F-*p*, 49.9
(a4b2_53) 1, $OC(O)NHC_6H_5$, 19.7
(a4b2_54) 1, $OC(O)NHC_2H_5$, 31.2
(a4b2_55) 1, OC(O)NH-*c*-C_5H_9, 13.1
(a4b2_56) 1, F, 7.3
18 1, H, 4.6
(a4b2_57) 2, H, 129

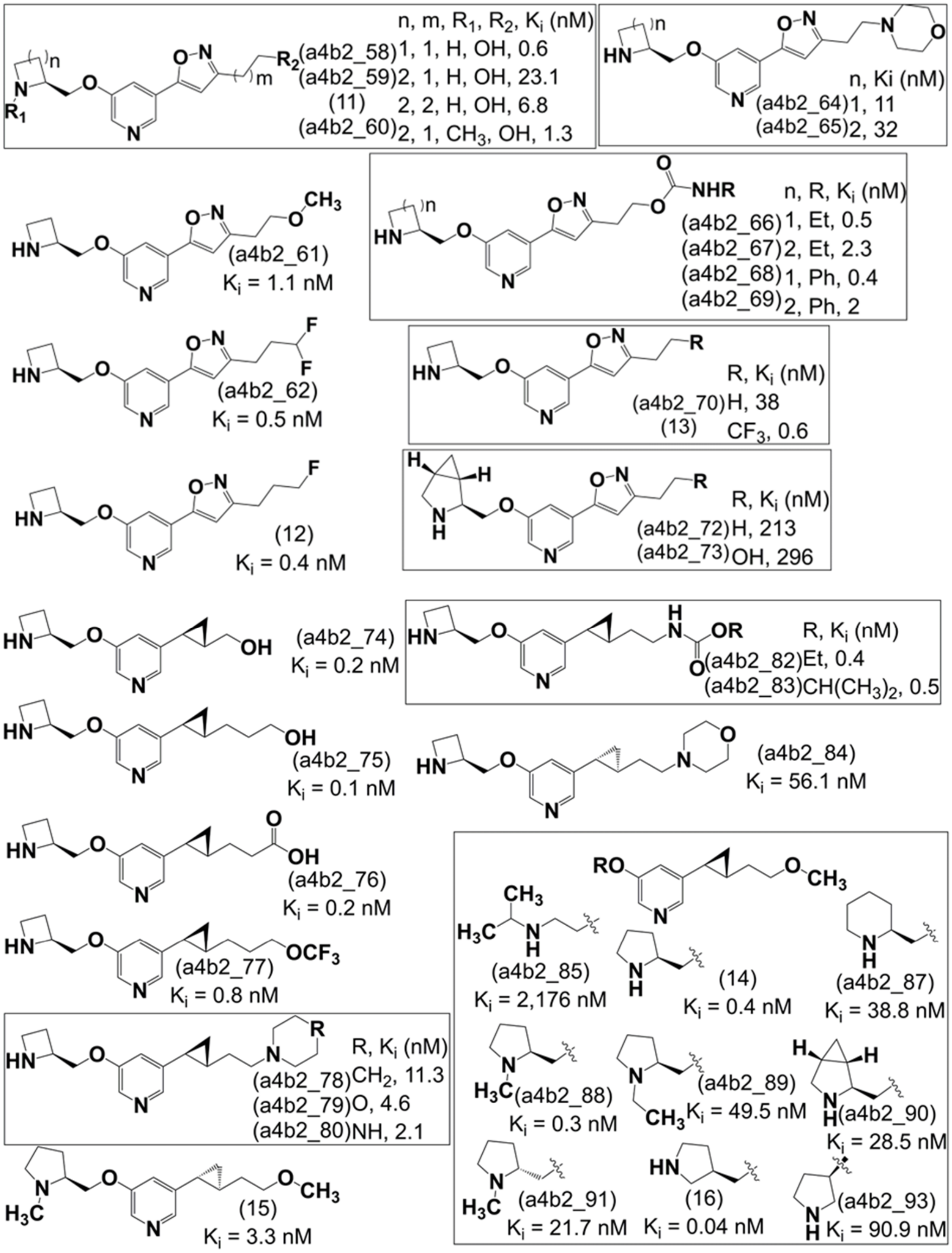
n, m, R1, R2, Ki (nM)
(a4b2_58) 1, 1, H, OH, 0.6
(a4b2_59) 2, 1, H, OH, 23.1
(11) 2, 2, H, OH, 6.8
(a4b2_60) 2, 1, CH3, OH, 1.3
n, Ki (nM)
(a4b2_64) 1, 11
(a4b2_65) 2, 32
(a4b2_61)
Ki = 1.1 nM
n, R, Ki (nM)
(a4b2_66) 1, Et, 0.5
(a4b2_67) 2, Et, 2.3
(a4b2_68) 1, Ph, 0.4
(a4b2_69) 2, Ph, 2
(a4b2_62)
Ki = 0.5 nM
R, Ki (nM)
(a4b2_70) H, 38
(13) CF3, 0.6
(12)
Ki = 0.4 nM
R, Ki (nM)
(a4b2_72) H, 213
(a4b2_73) OH, 296
(a4b2_74)
Ki = 0.2 nM
R, Ki (nM)
(a4b2_82) Et, 0.4
(a4b2_83) CH(CH3)2, 0.5
(a4b2_75)
Ki = 0.1 nM
(a4b2_84)
Ki = 56.1 nM
(a4b2_76)
Ki = 0.2 nM
(a4b2_77)
Ki = 0.8 nM
R, Ki (nM)
(a4b2_78) CH2, 11.3
(a4b2_79) O, 4.6
(a4b2_80) NH, 2.1
(15)
Ki = 3.3 nM
(a4b2_85)
Ki = 2,176 nM
(14)
Ki = 0.4 nM
(a4b2_87)
Ki = 38.8 nM
(a4b2_88)
Ki = 0.3 nM
(a4b2_89)
Ki = 49.5 nM
(a4b2_90)
Ki = 28.5 nM
(a4b2_91)
Ki = 21.7 nM
(16)
Ki = 0.04 nM
(a4b2_93)
Ki = 90.9 nM

All 93 ligands structures and their K_i used for dbCICA analysis of α7 are shown in Supplementary figure 3.

Figure S3. - Chemical structures of the 93 ligands used to construct the dbCICA models for the α7 subtype nAChRs.

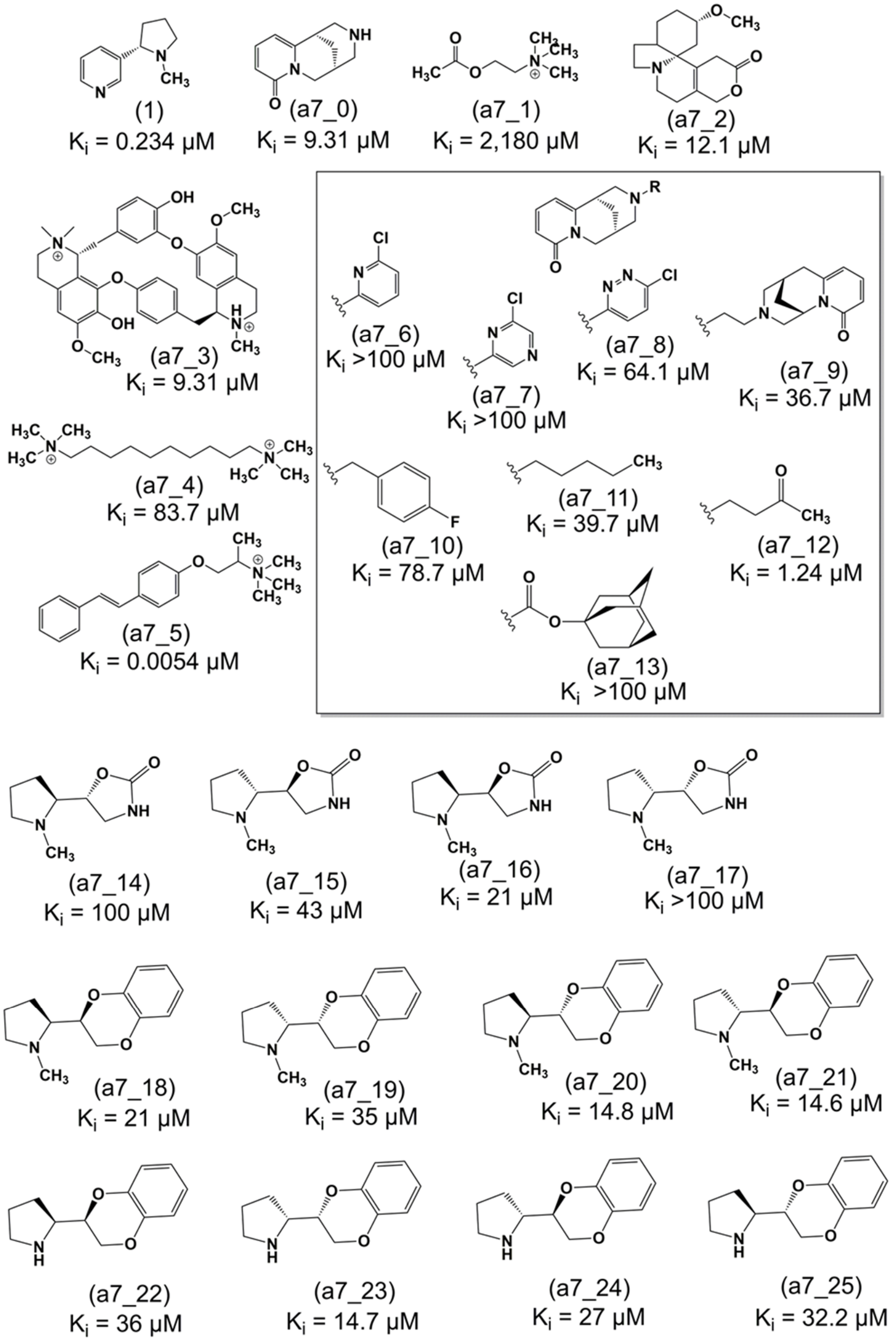

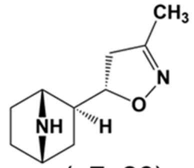

(a7_26)
K_i = 32 µM

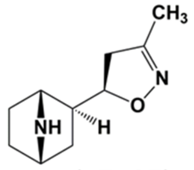

(a7_27)
K_i = 40 µM

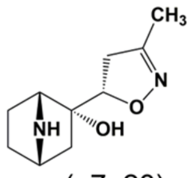

(a7_28)
K_i = 77 µM

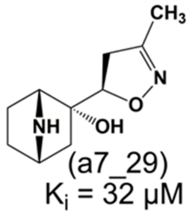

(a7_29)
K_i = 32 µM

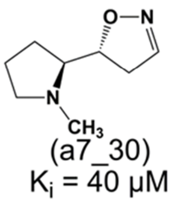

(a7_30)
K_i = 40 µM

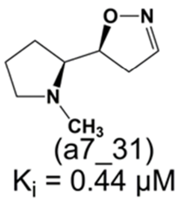

(a7_31)
K_i = 0.44 µM

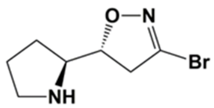

(a7_32)
K_i = 35 µM

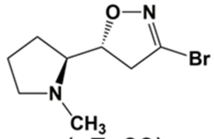

(a7_33)
K_i = 43 µM

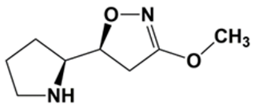

(a7_34)
K_i = 11.3 µM

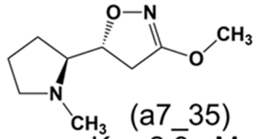

(a7_35)
K_i = 2.9 µM

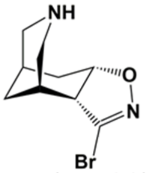

(a7_36)
K_i = 28.5 µM

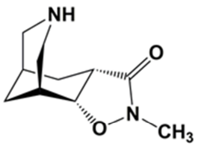

(a7_37)
K_i = 9.5 µM

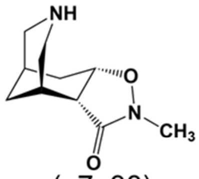

(a7_38)
K_i >100 µM

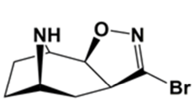

(a7_39)
K_i = 20 µM

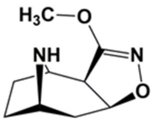

(a7_40)
K_i = 68 µM

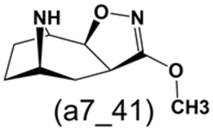

(a7_41)
K_i = 28.5 µM

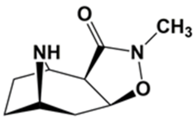

(a7_42)
K_i = 28.2 µM

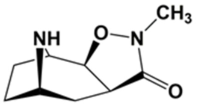

(a7_43)
K_i >100 µM

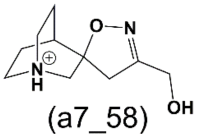
(a7_58)
K_i = 1.45 μM

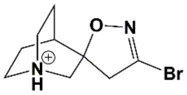
(a7_59)
K_i = 0.02 μM

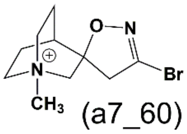
(a7_60)
K_i = 0.11 μM

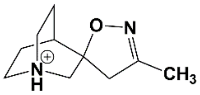
(a7_61)
K_i = 0.10 μM

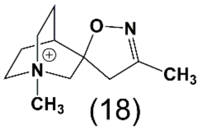
(18)
K_i = 0.76 μM

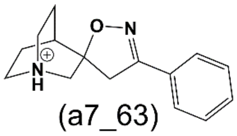
(a7_63)
K_i = 0.02 μM

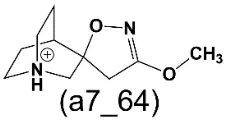
(a7_64)
K_i = 0.01 μM

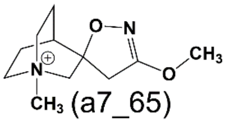
(a7_65)
K_i = 0.09 μM

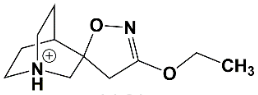
(19)
K_i = 0.19 μM

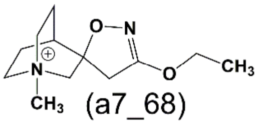
(a7_68)
K_i = 0.44 μM

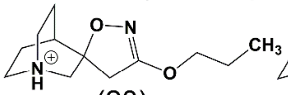
(20)
K_i = 0.07 μM

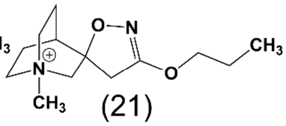
(21)
K_i = 0.83 μM

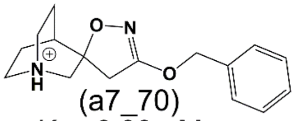
(a7_70)
K_i = 0.33 μM

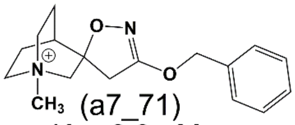
(a7_71)
K_i = 3.3 μM

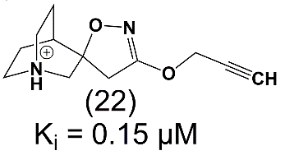
(22)
K_i = 0.15 μM

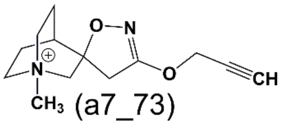
(a7_73)
K_i = 4.25 μM

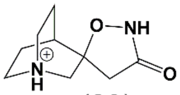
(23)
K_i = 5.7 μM

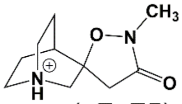
(a7_75)
K_i = 0.25 μM

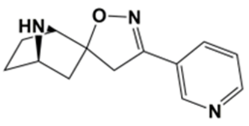

(a7_76)
K_i = 41 μM

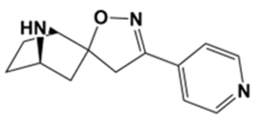

(a7_77)
K_i = 22 μM

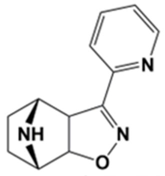

(a7_78)
K_i >100 μM

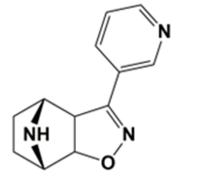

(a7_79)
K_i = 32 μM

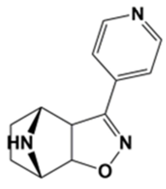

(a7_80)
K_i >100 μM

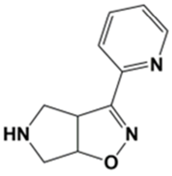

(a7_81)
K_i >100 μM

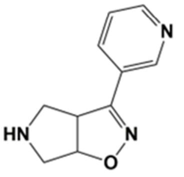

(a7_82)
K_i >100 μM

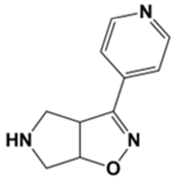

(a7_83)
K_i >100 μM

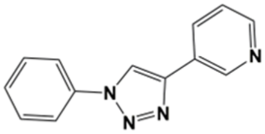

(a7_84)
K_i = 100 μM

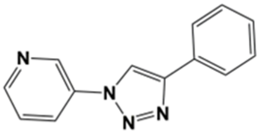

(a7_85)
K_i >100 μM

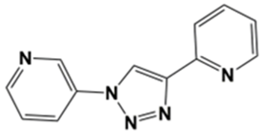

(24)
K_i = 6.5 μM

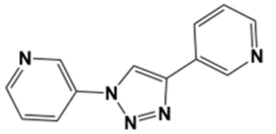

(a7_87)
K_i >100 μM

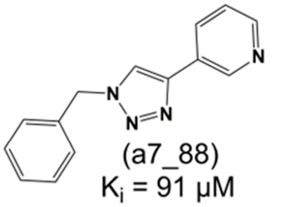

(a7_88)
K_i = 91 μM

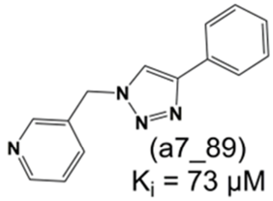

(a7_89)
K_i = 73 μM

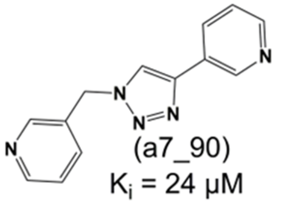

(a7_90)
K_i = 24 μM

ROC curves for studied nAChRs are shown in supplementary figure 4. The ROC curve values are AUC of 0.78 and 0.86, AAC of 0.83 and 0.83, and YA of 0.25 and 0.13 for nAChRs_α7_1 and nAChRs_α4β2_1 dbCICA, respectively.

Figure S4. – ROC curves of $(\alpha4)_2(\beta2)_3$ (a) and α7 (b) nAChRs pharmacophore maps validation.

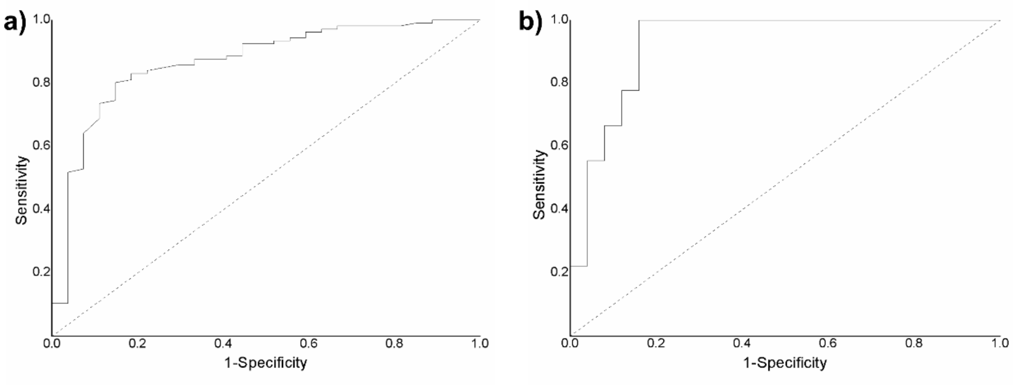

Supplement: Supplementary file 1 [file molecules-27-08236-s001.zip › molecules-1994877-supplementary.pdf]
